# Supplementary material for: Unravelling reference bias in ancient DNA datasets
Source: Bioinformatics. 2024 Jul 3;40(7):btae436. doi: 10.1093/bioinformatics/btae436 (PMC11254355; doi:10.1093/bioinformatics/btae436)
Supplement: btae436_Supplementary_Data [file btae436_supplementary_data.zip › S1-S4_data_plots.zip!/S1-S4_data/rhino_sim_plots.zip!/rhino_sim_plots/local_bowtie_rhino_div2_50end_by_MQ.pdf]

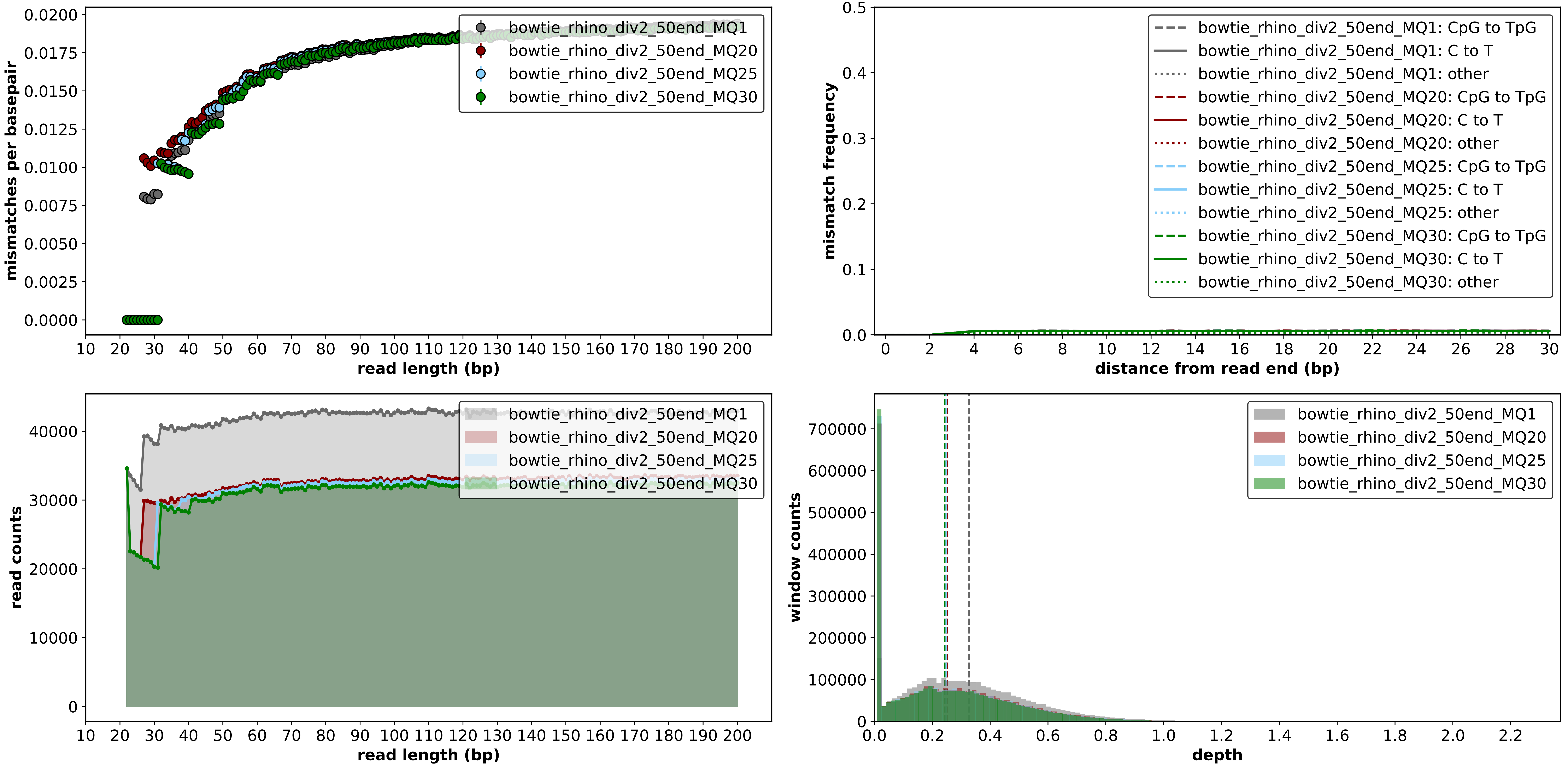

mismatches per basepair
0.0200
0.0175
0.0150
0.0125
0.0100
0.0075
0.0050
0.0025
0.0000
10 20 30 40 50 60 70 80 90 100 110 120 130 140 150 160 170 180 190 200
read length (bp)
bowtie_rhino_div2_50end_MQ1
bowtie_rhino_div2_50end_MQ20
bowtie_rhino_div2_50end_MQ25
bowtie_rhino_div2_50end_MQ30
mismatch frequency
0.5
0.4
0.3
0.2
0.1
0.0
0 2 4 6 8 10 12 14 16 18 20 22 24 26 28 30
distance from read end (bp)
bowtie_rhino_div2_50end_MQ1: CpG to TpG
bowtie_rhino_div2_50end_MQ1: C to T
bowtie_rhino_div2_50end_MQ1: other
bowtie_rhino_div2_50end_MQ20: CpG to TpG
bowtie_rhino_div2_50end_MQ20: C to T
bowtie_rhino_div2_50end_MQ20: other
bowtie_rhino_div2_50end_MQ25: CpG to TpG
bowtie_rhino_div2_50end_MQ25: C to T
bowtie_rhino_div2_50end_MQ25: other
bowtie_rhino_div2_50end_MQ30: CpG to TpG
bowtie_rhino_div2_50end_MQ30: C to T
bowtie_rhino_div2_50end_MQ30: other
read counts
40000
30000
20000
10000
0
10 20 30 40 50 60 70 80 90 100 110 120 130 140 150 160 170 180 190 200
read length (bp)
bowtie_rhino_div2_50end_MQ1
bowtie_rhino_div2_50end_MQ20
bowtie_rhino_div2_50end_MQ25
bowtie_rhino_div2_50end_MQ30
window counts
700000
600000
500000
400000
300000
200000
100000
0
0.0 0.2 0.4 0.6 0.8 1.0 1.2 1.4 1.6 1.8 2.0 2.2
depth
bowtie_rhino_div2_50end_MQ1
bowtie_rhino_div2_50end_MQ20
bowtie_rhino_div2_50end_MQ25
bowtie_rhino_div2_50end_MQ30
